# Diagnostic value of tumor-fascia relationship in superficial soft tissue masses on magnetic resonance imaging

**DOI:** 10.1371/journal.pone.0209642

**Published:** 2018-12-31

**Authors:** Tadashi Iwai, Manabu Hoshi, Naoto Oebisu, Masanari Aono, Masatugu Takami, Makoto Ieguchi, Hiroaki Nakamura

**Affiliations:** 1 Department of Orthopedic Surgery, Osaka City University Graduate School of Medicine, Osaka-city, Osaka, Japan; 2 Department of Orthopedic Surgery, Osaka City General Hospital, Osaka-city, Osaka, Japan; 3 Department of Orthopedic Surgery, Hanwasumiyoshi General Hospital, Osaka-city, Osaka, Japan; 4 Department of Orthopedic Surgery, Fuchu Hospital, Izumi-city, Osaka, Japan; University of Miami School of Medicine, UNITED STATES

## Abstract

**Purpose:**

Many surgeons participate in the management of superficial soft tissue masses, and a preoperative incorrect diagnosis frequently results in dismal oncological outcomes. The aim of this study was to identify distinguishing magnetic resonance imaging features between malignant and non-malignant lesions.

**Methods:**

The clinicopathological data for 219 patients (men 114; women 105) with superficial soft tissue masses treated from January 2007 to December 2016 in our institution were retrospectively analyzed. The median age at the first visit was 55.6 years (range 1–90 years). MRI findings of tumor size, margin, lobulation, intratumoral hemorrhage, peritumoral edema, and tumor-fascia relationship were compared with the final histological diagnosis and tumor grade.

**Results:**

Univariate analysis revealed significant relationships between histologically malignant lesions and tumor size ≥5 cm (p = 0.035), positive peritumoral edema (p = 0.031), and tumor-fascia relationship (p<0.001), but not margin (p = 0.107), lobulation (p = 0.071), and intratumoral hemorrhage (p = 0.17). In addition, using multivariate analysis, the tumor-fascia relationship (p<0.001) and tumor size were significant factors.

A significant correlation between tumor-fascia relationship and malignancy (p<0.001) was observed; such a relationship was, however, not observed for tumor grade (p = 0.43).

**Conclusions:**

Tumors measuring ≥5 cm and the tumor-fascia relationship on magnetic resonance imaging are highly indicative of malignancy. When superficial soft tissue masses cross the superficial fascia and form obtuse angles with the fascia, sarcoma should be considered. The tumor-fascia relationship can offer surgeons useful information regarding the status of superficial soft tissue masses.

## Introduction

Soft tissue sarcomas are rare malignancies, and constitute less than 1% of all malignant tumors [1] Most superficial soft tissue sarcomas occur in the extremities [2]. In general, tumors measuring >5 cm, those that continue to grow in size, and those that are painful and deep-seated are likely to be malignant [3]. Superficial masses include primary soft tissue sarcomas, metastatic tumors, benign tumors, and non-neoplastic lesions such as bursitis and ganglions. Magnetic resonance imaging (MRI) is the main imaging modality for evaluation of soft tissue tumors [4]. Although MRI contributes towards a specific pathological diagnosis or a narrow differential diagnosis on the basis of the signal intensity [5], its ability to distinguish between benign and malignant soft tissue tumors remains to be debated [6]. Moreover, little information regarding the MRI features of superficial soft tissue masses is available. The aim of our study was to identify characteristic factors for differentiation between malignant and non-malignant lesions, and to analyze the correlation between the tumor-fascia relationship and malignant tumors. Herein, we focused on the MRI features of subcutaneous soft tissue masses.

### Patients and methods

#### Data collection

We retrospectively reviewed the clinical data for 219 patients (114 male and 105 female; mean age at first visit 55.6 years [range 1–90 years]) with superficial soft tissue masses treated between January 2007 and December 2016 at the Department of Orthopedic Surgery, Osaka City University Hospital. The inclusion criterion was the detection of superficial soft tissue masses on MRI examination. All patients underwent MRI before echo-guided needle biopsy or primary excision biopsy, after which the pathological diagnosis was determined. We investigated clinical factors such as patient demographics, histological diagnosis, and anatomic location.

#### MRI analysis

**Fig 1:**
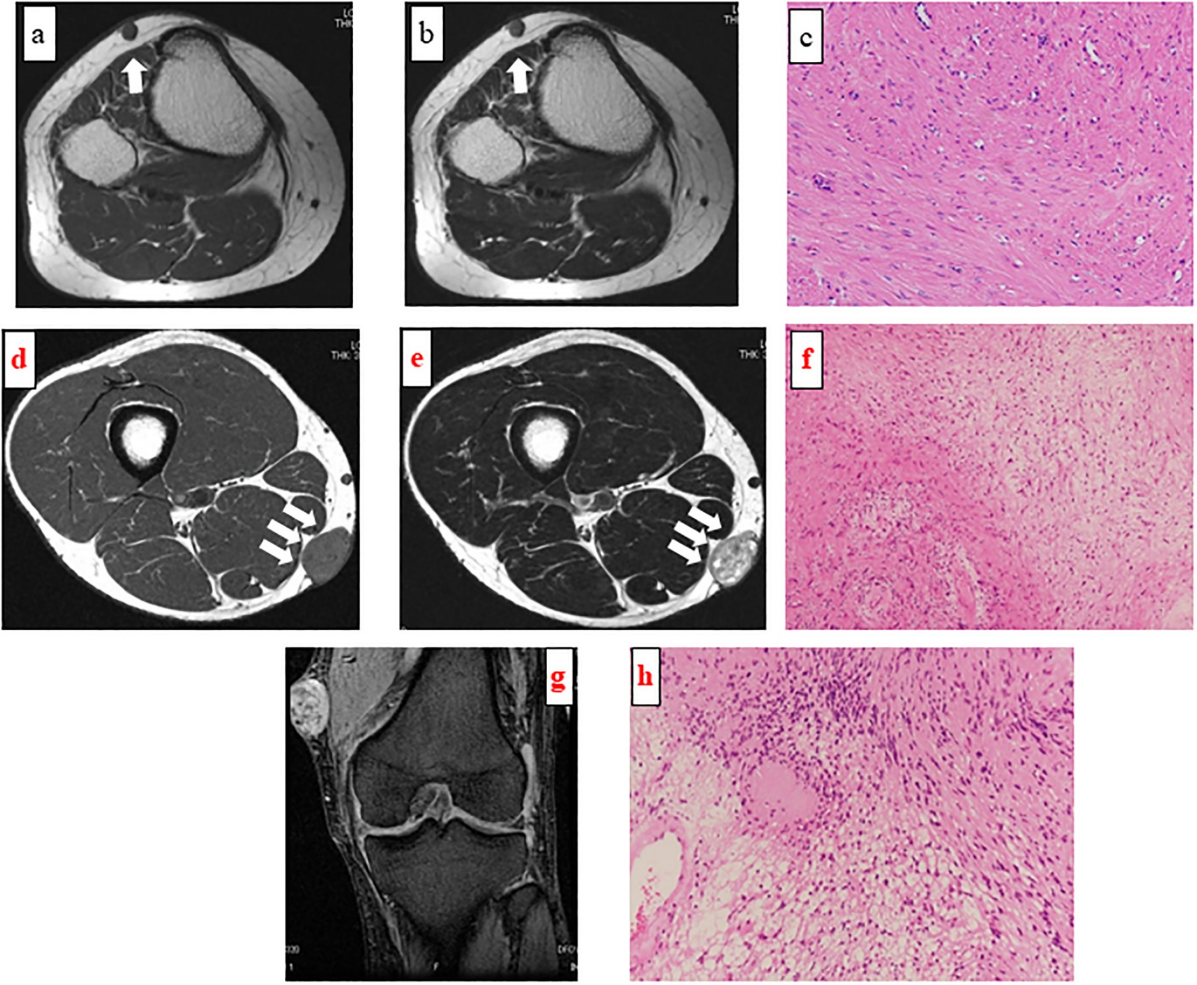
Magnetic resonance imaging findings of a superficial mass of the right lower leg in a 41-year-old woman. Axial T1-weighted (a) and T2-weighted (b) images showing that the lesion did not contact the superficial fascia (Group 1 per the Galant classification). Pathologic examination of the resected specimen confirmed angioleiomyoma (c; hematoxylin-eosin staining; magnification ×200).

**Fig 2:**
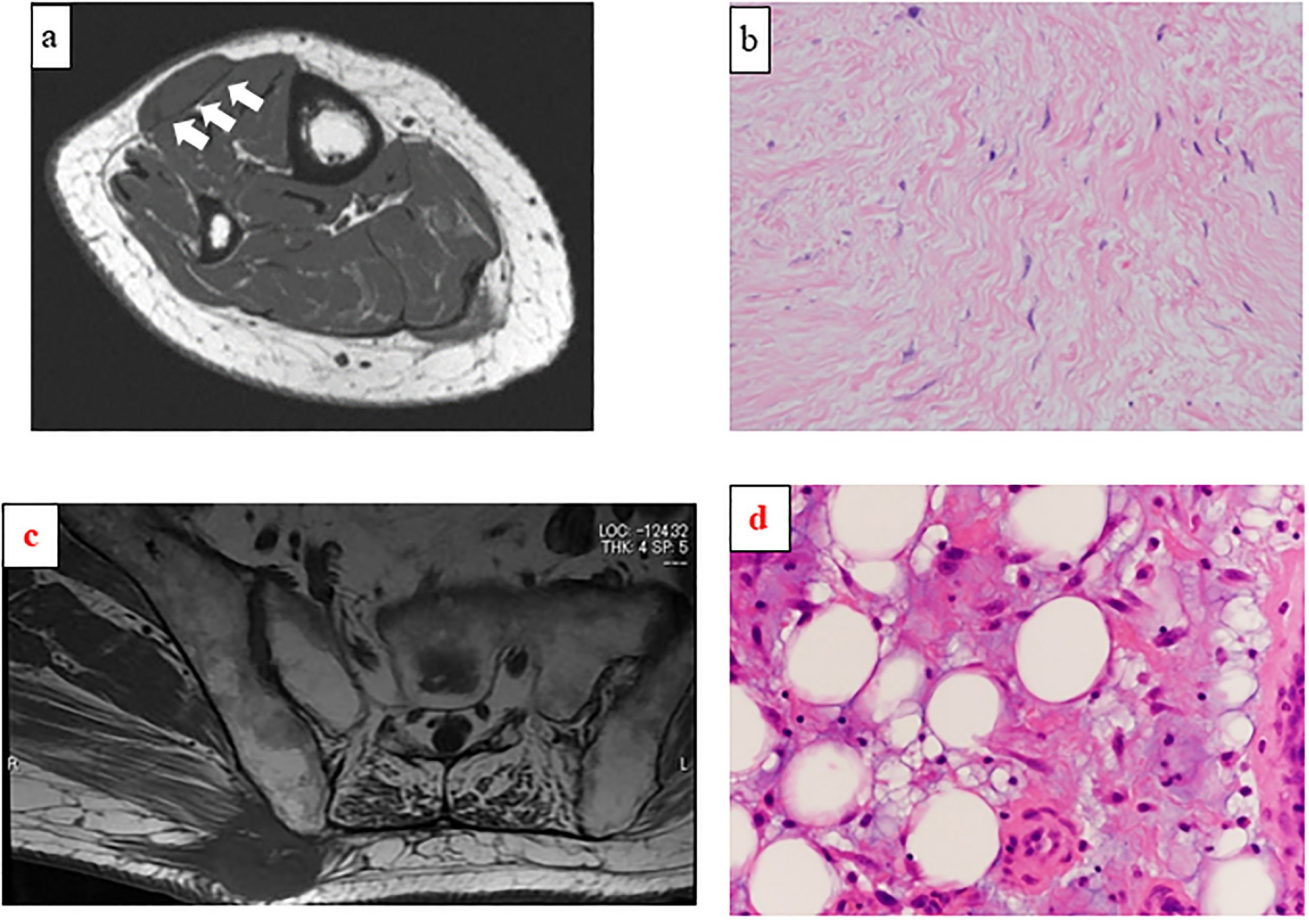
Magnetic resonance imaging findings of a superficial mass in the anterior part of the right lower leg in a 78-year-old woman. Axial T1-weighted image showing a well-defined lesion with wider contact with the fascia at obtuse angles (a; Group 4 per the Galant classification). Pathologic examination of the resected specimen confirmed low-grade fibromyxoid sarcoma (b; hematoxylin-eosin staining; magnification ×200).

For the purpose of the present study, a superficial soft tissue mass was defined on the basis of its location: cutaneous, subcutaneous or fascial, and overlying the muscle. T1 and T2-weighted images in the axial and coronal and/or sagittal planes were obtained for all patients. The MR information was assessed by two orthopedic oncologists. Differences were resolved by consensus. Lesion size was measured in longitudinal, anterior-posterior, and transverse dimensions. The maximal diameter of the lesion was then measured and recorded. Lesions were classified into two groups based on their size: <5 cm and ≥5 cm. MRI findings examined included tumor size, margin, lobulation, intratumoral hemorrhage, peritumoral edema, and tumor-fascia relationship based on the Galant classification [7]. Any contact or extension through the superficial fascia was also recorded. Galant et al. (1998) established an ordered graduation of the tumor-fascia relationship. Group 1 tumors did not contact the fascia (Fig 1); group 2 lesions slightly contacted the fascia, with acute angles between the tumor and the fascia (Fig 1); group 3 lesions had wider contact with the fascia at larger acute or right angles (Fig 1); group 4 tumors had even wider contact with the fascia at obtuse angles (Fig 2); and group 5 lesions were composed of lesions that crossed the superficial fascia (Fig 2). They also reported that obtuse angles (group 4) between a superficial tumor and the fascia or a tumor crossing the fascia (group 5) strongly suggested the presence of a malignant tumor (7). We also classified group 1, 2, and 3 lesions as non-malignant, and group 4 and 5 lesions as malignant.

#### Magnetic resonance imaging findings of a superficial mass of the right thigh in a 33-year-old man

Axial T1-weighted (d) and T2-weighted (e) images demonstrating a well-defined mass slightly contacting the fascia (Group 2 per the Galant classification). Pathologic examination of the resected specimen confirmed schwannoma (f; hematoxylin-eosin staining; magnification ×100).

#### Magnetic resonance imaging findings of a superficial mass of the left knee in a 62-year-old man

Coronal T2-weighted images demonstrating a well-defined lesion with wider contact with the fascia at a larger acute angle (Group 3 per the Galant classification) (g). Pathologic examination of the resected specimen confirmed schwannoma (h; hematoxylin-eosin staining; magnification ×200).

#### Magnetic resonance imaging findings of a superficial mass of the right buttock in a 79-year-old man

Axial T1-weighted image showing a low-density mass invading the superficial fascia (c; Group 5 per the Galant classification). Pathologic examination of the resected specimen confirmed myxoid liposarcoma (d; hematoxylin-eosin staining; magnification ×400).

#### Pathological analysis

All the specimens were examined by a pathologist specialized in sarcoma pathology and diagnosed according to the standard criteria for soft tissue sarcoma subtyping [8]. All the soft tissue sarcomas were graded according to the three-tier French system [9,10], with grade 2 and 3 tumors considered to be high-grade. The patients were divided into two distinct groups based on the final histological diagnosis: those with malignant tumors and those with non-malignant tumors (benign tumors or non-neoplastic masses). Then, the final pathological diagnosis was compared. We focused on the tumor-fascia relationship, wherein we compared the tumor-fascia relationship between metastatic tumors and sarcomas and assessed the association between the tumor-fascia relationship and tumor grade. The present study was approved by the Institutional Review Board of Osaka City University Graduate School of Medicine.

#### Statistical analysis

Fisher’s exact probability test was performed to conduct a statistical comparison of the two groups. Univariate analysis was performed by means of the Fisher’s exact probability test. Multivariate analysis was performed by the binomial logistic regression analysis. The statistical analysis was performed using Excel statistics software (version 2015; SSRI Co., Ltd) for Windows. *P* values <0.05 were considered statistically significant.

## Results

Histopathologically, 65 cases of malignant tumors and 154 cases of non-malignant lesions were recorded. The superficial soft tissue masses were located in the lower limbs in 77 patients, trunk in 78 patients, and upper limbs in 64 patients. In terms of tumor grade, 46 cases of high-grade tumors were detected and 10 cases of low-grade tumors. Regarding tumor size, 86 cases involved a tumor measuring >5 cm, of which 33 (38.4%) cases involved malignant tumors. The mean size of all masses was 4.7±3.6 cm. The mean size of malignant tumors was 5.7±3.7 cm, while that of non-malignant masses was 4.2±3.5 cm (Table 1).

**Table 1:** Patients demographic data.

Factors		Number
Male		114
Female		105
Age (years old)		55.6±18.9
Malignant		65
Non-malignant		154
Tumor size (cm)		
Total		4.7±3.6
Malignant		5.8±3.7
Non-malignant		4.2±3.5
Anatomical location		
Extremities	Upper arm	12
	Elbow	6
	Forearm	7
	Hand	39
	Thigh	27
	Knee	9
	Lower leg	20
	Foot	21
Trunk	Chest	11
	Shoulder	17
	Back	38
	Abdomen	11
	Head	1
Tumor grade	High	46
	Low	10

After investigation of the MRI features of superficial soft tissue masses, univariate analysis revealed that significant associations were observed between malignancy and tumor size (p = 0.035), peritumoral edema (p = 0.031), and the tumor-fascia relationship (p<0.001), but not between malignancy and margin (p = 0.107), lobulation (p = 0.071), and intratumoral hemorrhage (p = 0.17). In addition, using multivariate analysis, the tumor-fascia relationship (p<0.001) and tumor size (p = 0.047) were identified as significant factors of malignancy (Table 2).

**Table 2:** MRI features in superficial soft tissue masses.

**Variable (Univariate analysis)**		**Non-malignant**	**Malignant**	**p-value**
Tumor Size	<5 cm	100	32	0.035
	≧5 cm	54	33	
Margin	Well defined	113	40	0.107
	Irregular	41	25	
Lobulation	Positive	57	33	0.071
	Negative	97	32	
Intratumoral hemorrhage	Positive	53	29	0.17
	Negative	101	36	
Peritumoral edema	Positive	47	30	0.031
	Negative	107	35	
Tumor-fascia relationship (Galant classification)	1·2·3	107	21	<0.001
	4·5	47	44	
Variable (Multivariate analysis)	B	S.E.	OR (95%CI)	p-value
Tumor Size	0.74	0.37	2.10 (1.01–4.37)	0.047
Margin	-0.51	0.44	0.60 (0.25–1.43)	0.248
Lobulation	0.41	0.39	1.51 (0.71–3.22)	0.283
Intratumoral hemorrhage	0.12	0.41	1.13 (0.50–2.54)	0.77
Peritumoral edema	0.26	0.38	1.29 (0.62–2.72)	0.497
Tumor-fascia relationship (Galant classification)	1.77	0.39	5.86 (2.74–12.5)	<0.001

B, Regression coefficient; S.E., Standard error; OR, Odds ratio; CI, Confidence intervals

A well-defined margin was recorded in 40 out of 65 cases of malignant tumors; the remaining 25 cases demonstrated irregular margins. However, an irregular margin was recorded in 41 of 154 cases of benign tumors. Moreover, 57 of the 154 non-malignant masses showed a lobular mass on MRI; 29 of the 65 malignant tumors showed intratumoral hemorrhage, while 101 of the 154 non-malignant masses did not involve hemorrhage. We also found that 107 of the 154 benign masses had no edema around the tumor, whereas 30 of the 65 malignant tumors had edema around the tumor.

Histopathologically, the most common type of malignant tumors observed were pleomorphic liposarcoma (11 cases, 17%), myxofibrosarcoma (6 cases, 9.4%), and leiomyosarcoma (5 cases, 7.7%). The three most commonly observed non-malignant tumors were lipoma (43 cases, 28%), schwannoma (22 cases, 14%), and giant cell tumor of the tendon sheath (22 cases, 14%).

Regarding the tumor-fascia relationship, the total number of group 1, 2, and 3 lesions was 128; 107 were non-malignant and 21 were malignant. The total number of group 4 and 5 lesions was 91; 47 were benign and 44 were malignant (Table 3).

**Table 3:** The relationship of each histology to superficial fascia based on Galant classification.

	Number	Galant classification				
		1	2	3	4	5
*Malignant lesions*						
Pleomorphic liposarcoma	11				4	7
Myxofibrosarcoma	6	1		1	2	2
Leiomyosarcoma	5			1	1	3
Dedifferentiated liposarcoma	5			1	2	2
Metastatic tumor	5	2			1	2
Malignant lymphoma	4	2	1	1		
Myxoid liposarcoma	4				3	1
Epithelioid sarcoma	4	1				3
Atypical lipomatous tumor	4			3	1	
Undifferentiated pleomorphic sarcoma	4	1				3
Solitary fibrous tumor	2			1		1
Malignant peripheral nerve sheath tumor	2			1		1
Synovial sarcoma	2	1		1		
Clear cell sarcoma	1					1
Ewing sarcoma	1					1
Dermatofibrosarcoma	1				1	
Ossifying fibromyxoid tumor	1	1				
Inflammatory myofibroblastic tumor	1	1				
Low grade fibromyxoid sarcoma	1				1	
Alveolar soft part sarcoma	1					1
Total number of lesions	65	10	1	10	16	28
*Non-malignant*						
Lipoma	43	3	4	35	1	
Schwannoma	22		3	12	5	2
Giant cell tumor of tendon sheath	22			11	5	6
Atheroma	18	3	3	8	4	
Angioleiomyoma	8	1		4	3	
Fibromatosis	8	1	1	1	4	1
Angioma	7		1	2	3	1
Bursitis	4			2	2	
Ganglion	3			1	2	
Neurofibroma	3				2	1
Leiomyoma	2		1			1
Hematoma	2		1	1		
Dermatofibroma	1	1				
Reactive lymphoid hyperplasia with infiltration of eosinophils	1					1
Fibrolipoma	1			1		
Pilomatricoma	1	1				
Epithelioid granuloma	1					1
Kimura disease	1			1		
Spindle cell lipoma	1			1		
Calcifying aponeurotic fibroma	1				1	
Subcutis	1	1				
Pleomorphic lipoma	1			1		
Granuloma	1	1				
Tophus	1				1	
Total number of lesions	154	12	14	81	33	14

We added the number of malignant lymphomas to that of the metastatic tumor because the locations of the malignant lymphomas in this study were not as considerable as the primary tumor (Table 4).

**Table 4:** Tumor-fascia relationship of superficial malignant tumors.

	Galant classification		p-value
	1·2·3	4·5	
Malignant tumor			
Sarcoma	15	41	0.048
Metastasis	6	3	
Tumor grade of sarcoma			
High grade	11	35	0.43
Low grade	4	6	

A significant relationship was observed between the tumor-fascia relationship and metastatic tumors and sarcomas (p = 0.048): 41 out of 56 sarcomas were classified into group 4 or 5, while 6 out of 9 metastatic tumors including malignant lymphomas were classified into group 1, 2, or 3 (Table 4). However, no significant association between malignancy and tumor grade was observed (p = 0.43). According to tumor grade, 35 out of 46 high-grade sarcomas were diagnosed as group 4 or 5 lesions. However, 6 out of 9 low-grade sarcomas were also classified into group 4 or 5 (Table 4).

## Discussion

Superficial soft tissue sarcomas present different characteristics from their deep-seated counterparts. Classically, a soft tissue mass should be suspected as malignant, if it measures >5 cm and its location is deep within the fascia. Soft tissue sarcomas uncommonly manifest superficially to the fascia [11]. However, superficial soft tissue masses are relatively common in general clinical orthopedic practice. The incidence of sarcomas is rare, being estimated at approximately 3 cases per 100000 population annually in Japan [12], and superficial soft tissue sarcomas account for 20% of all soft tissue sarcomas [13]. Most superficial soft tissue sarcomas occur in the extremities, especially in the thigh [2]. Clinically superficial soft tissue sarcomas occasionally tend to be resected via inappropriate techniques [14–16]. Surgeons who specialize in fields other than surgical oncology do participate in the management of soft tissue masses, especially when the tumor is located subcutaneously, and tend to perform simple tumor excisions without any concern for malignancy [17]. Therefore, because these malignant tumors are inappropriately treated, their prognosis becomes less favorable. While there does exist plenty of evidence regarding deep-seated soft tissue sarcomas, only a few studies have been devoted to this special subtype. Incorrect diagnosis and treatment of soft tissue sarcomas frequently results in tumor recurrence. To avoid dismal oncological outcomes of superficial soft tissue sarcomas, a separate and detailed analysis of this group should therefore be performed, especially to ensure accurate diagnoses.

Many problems concerning conventional diagnostic modalities were resolved by the introduction of MRI. MRI can predict the quality of the tumor and often helps to narrow the differential diagnosis [18]. Lipomas, hemangiomas, cysts, hematomas, vascular malformation, and pigmented villonodular synovitis (giant cell tumor of the tendon sheath) are relatively easy to diagnose from MRI information. Berquist et al. demonstrated that MRI could distinguish benign lesions from malignant lesions with an average sensitivity of 94%, specificity of 90%, and accuracy of 90%, while Kransdorf et al. showed that only 24% of soft tissue tumors had a specific feature that could be observed on MRI [18,19]. The accuracy of MRI in distinguishing between benign and malignant soft tissue tumors has always been controversial.

In the present study, the feature highly associated with malignancy was the relationship between the mass and the superficial fascia; additionally, the size of the mass and the presence of peritumoral edema were also significant factors. On the basis of the MRI features of superficial soft tissue masses documented in previous reports, Galant et al. proposed that the relationship of a mass with the superficial fascia is important for predicting malignancy [7]. Calleja et al. found that lobulation, hemorrhage, fascial edema, necrosis, skin thickening, and skin contact are significant factors indicative of malignancy [1]. It is also well recognized that tumors measuring >5 cm is an important factor to differentiate between malignant and benign masses in both the deep and superficial subset [3]; furthermore, Lachenmayer et al. showed that most superficial soft tissue sarcomas were ≥5 cm [2].

Galant et al. [7] focused on the tumor-fascia relationship in superficial soft tissue tumors to differentiate between malignant tumors, and proposed that subcutaneous lesions crossing the superficial fascia (group 5 per the Galant classification) and forming obtuse angles with the fascia (group 4) are more likely to be malignant than those without these signs. The result of the present study proved to be significantly consistent with this classification (p<0.01). Galant et al. proposed that the fascia represents the deep limit of the subcutaneous compartment and constitutes a natural barrier to tumor spread [7]. Kawaguchi et al. also indicated that the superficial fascia can be classified as a thin barrier and considered a surgical safety margin for soft tissue sarcomas [20]. Benign tumors usually expand to push out surrounding tissues including the fascia but infrequently infiltrate the tissue, resulting in acute angles between the tumor and the fascia. On the other hand, malignant tumors tend to extend along the fascia and often invade or cross the fascia. The tumor-fascia relationship in superficial soft tissue tumors can demonstrate the local aggressiveness of the malignant tumor. Moreover, the tumor-fascia relationship seems to differ among primary and metastatic tumors.

The tumor-fascia relationship is not always reliable in terms of differentiating between malignant and non-malignant lesions. False negative results can be obtained, and can thus hinder an accurate diagnosis and appropriate treatment. Surgeons should be aware of the different tumor characteristics that could yield false negative results. Interestingly, 6 cases of malignant lymphoma and metastatic tumors were classified into group 1, 2, or 3 lesions. Moreover, there was a significant difference based on the tumor-fascia relationship between sarcomas and metastases. This result suggested that metastatic tumors including malignant lymphomas tend to reveal different MRI features from those of primary soft tissue sarcomas in superficial areas. Primary soft tissue sarcomas are likely to show more aggressive features in superficial regions than metastatic tumors.

In primary soft tissue sarcomas, it is expected that high-grade sarcomas would demonstrate locally aggressive behaviors, such as those lesions belong to group 4 and 5 of the Galant classification. However, we could not find a significant association between the tumor-fascia relationship and tumor grade (Table 4). Thus, tumor grade did not influence tumor-fascia relationship.

In the present study of superficial soft tissue tumors, the most common histopathology observed was liposarcoma (36.9%), myxofibrosarcoma (9.2%), and leiomyosarcoma (7.7%). In a recent analysis of 367 superficial soft tissue sarcomas, the French Sarcoma Group reported that the most frequent lesions observed were unclassified sarcomas (24.3%) and leiomyosarcomas (22.3%), followed by dermatofibrosarcoma protuberans (17.4%), angiosarcomas (14.4%), and myxofibrosarcomas (9%) [21]. Another study demonstrated that the most commonly reported superficial sarcomas were liposarcomas (30%), malignant fibrous histiocytomas (23%), and leiomyosarcomas (14%) [2]. These results were consistent with the general incidence reported in the registry of soft tissue sarcomas [22].

This study had several limitations such as a small number of enrolled patients and the retrospective nature of the study, Moreover, this study was performed in a single Japanese institution. Our results should be validated in a study with a larger number of patients involving multiple centers. Second, the backgrounds of the patients included various stages of malignant soft tissue tumors, various types of histology, various tumor grades, and various locations. Especially, the frequency of pleomorphic liposarcoma during data collection was large. In general, it only accounted for 5–10% of liposarcoma cases [23]. However, all specimens from the resected tissues were diagnosed by two pathologists with specialized training and expertise in sarcoma pathology, using standard diagnostic criteria for bone and soft tissue sarcoma subtyping [8]. Moreover, the number of lipomas was not only high, but the size of the lipomas was also enormous. Lipomas could usually be easily diagnosed with proper application of T1, T2, and fat-suppressed sequences. Thus, selection bias might have occurred in this study. Third, our study did not include contrast-enhanced MRI features. As we could not analyze the difference between edema and necrosis, we excluded necrosis from among evaluation criteria of MRI features. Moreover, a superficial soft tissue mass was defined on the basis of its location: cutaneous, subcutaneous or fascial, and overlying the muscle. However, there are no concrete criteria, as we evaluated the location of lesions by investing each MRI images based on Galant classification [7]; this is likely to be a major limitation of our study. Fourth, we did not conduct a pathologic confirmation of the presence of superficial fascia invasion. Fifth, the number of metastatic tumors in our study was rather small, thus, it may be controversial whether or not primary soft tissue sarcomas are likely to show more aggressive features in superficial regions than metastatic tumors.

In conclusion, tumor size and the tumor-fascia relationship observed on MRI were closely associated with malignancy of superficial soft tissue masses. When primary superficial soft tissue sarcomas cross the superficial fascia and form obtuse angles with the fascia, malignancy should be suspected; metastatic tumors did not present these features. However, no significant correlation between the tumor-fascia relationship and tumor grade was observed. The tumor-fascia relationship can offer orthopedic surgeons useful information regarding a preoperative diagnosis of superficial soft tissue masses.

### Ethical approval

The present study was approved by the Institutional Review Board of Osaka City University Graduate School of Medicine and was performed in accordance with the ethical standards laid down in the Declaration of Helsinki. The persons included in the study provided informed consent prior to their inclusion in the study.

## Supporting information

S1 Table Anonymized minimal data set. (XLSX)
